# Supplementary material for: Field-free spin-orbit torque switching assisted by in-plane unconventional spin torque in ultrathin [Pt/Co]N
Source: Nat Commun. 2023 Jul 4;14:3932. doi: 10.1038/s41467-023-39649-1 (PMC10319890; doi:10.1038/s41467-023-39649-1)
Supplement: Supplementary file 1 — Supplementary Information File [file 41467_2023_39649_MOESM1_ESM.docx]

**Supplementary Information**

**Field-free spin-orbit torque switching assisted by in-plane unconventional spin torque in ultrathin [Pt/Co]_N_**

Fen Xue^1*^, Shy-Jay Lin^2^, Mingyuan Song^2^, William Hwang^1^, Christoph Klewe^3^, Chien-Min Lee^2^, Emrah Turgut^2^, Padraic Shafer^3^, Arturas Vailionis^4,5^, Yen-Lin Huang^2^, Wilman Tsai^6^, Xinyu Bao^2^, and Shan X. Wang^1,6^*

^1^Department of Electrical Engineering, Stanford University, Stanford, California 94305, USA

^2^Taiwan Semiconductor Manufacturing Company, Hsinchu, Taiwan

^3^Advanced Light Source, Lawrence Berkeley National Laboratory, Berkeley, California 94720, USA

^4^Stanford Nano Shared Facilities, Stanford University, Stanford, CA 94305, USA

^5^Department of Physics, Kaunas University of Technology, LT-51368 Kaunas, Lithuania

^6^Department of Materials Science and Engineering, Stanford University, Stanford, California 94305, USA

*Corresponding authors: [fenx@stanford.edu](mailto:fenx@stanford.edu) and [sxwang@stanford.edu](mailto:sxwang@stanford.edu)

**Supplementary Methods**

**Angular-dependent second harmonic Hall (SHH) measurement for SOT characterization.** The angular-dependent second harmonic Hall (SHH) measurement was performed on Hall-bar devices of samples [Pt(1)/Co(*t*_Co_)]_5_/Mg(2)/CoFeB(2.5)/MgO(1.5)/Ta(2) deposited on either MgO single crystal substrates or Si/SiO_2_ substrates. An ac current with an amplitude of 2mA and a frequency of 1.33 kHz was injected into the 10μm-width current channel of the Hall bar; and the current channels with different widths have the same injected current density. The first (*V*_H,1ω_) and second (*V*_H,2ω_) harmonic Hall voltages along the transverse direction were measured using lock-in amplifiers as a function of the angle *ϕ* of the in-plane external magnetic field *H*_ext_ with an amplitude of 20 to 700 mT. The corresponding Hall resistances *R*_H,1ω_ and *R*_H,2ω_ vs. ϕ of (SRT1 or SRT2)/Mg(2)/CoFeB(2.5)/MgO(1.5)/Ta(2) samples measured at 2 mA and 200 mT are shown in Fig. 2 in the main manuscript. The *R*_H,2ω_ curve of the SRT2-based sample in Fig. 2h is a representative curve for most [Pt(1)/Co(*t*_Co_)]_5_-based samples which shows negligible unconventional spins deposited on either Si/SiO_2_ substrate or MgO single crystals. Here, *R*_H,1ω_ vs. ϕ can extract out the Hall-effect related parameters from

$R_{H,1\omega}=\frac{R_{A}}{2}\cos\theta+\frac{R_{P}}{2}\sin^{2}\theta\sin2\varphi$ (1)

where, θ and ϕ are the out-of-plane and in-plane magnetization angles, respectively. *R*_A_ is anomalous Hall resistance, estimated by sweeping the magnetic field along the out-of-plane direction. *R*_P_ is planar Hall resistance fitted by *R*_H,1ω_ vs. ϕ in Supplementary Eq. (1). In the SRT1-based, SRT2-based, and pure Pt-based samples, *R*_A_ is respectively 0.51 Ω, 1.38 Ω, and 0.28 Ω, and *R*_P_ is respectively 0.033 Ω, 0.095 Ω, and 0.7 Ω. From *ϕ*-dependent fitting of *R*_H,2ω_, we can obtain the spin-orbit effective fields associated with the damping-like torques and field-like torques from different polarized spins, and thereby calculate the spin efficiencies [1-4]:

$R_{H,2\omega}=R_{AD,x}\sin\varphi+R_{AD,y}\cos\varphi+R_{AD,z}cos2\varphi+R_{FL,x}cos2\varphi\sin\varphi+R_{FL,y}cos2\varphi\cos\varphi+R_{FL,z}$

(2)

with

$R_{AD,x}={\frac{1}{2}R}_{A}\frac{H_{AD,x}}{H_{ext}+H_{k}}$ (3)

$R_{AD,y}={\frac{1}{2}R}_{A}\frac{H_{AD,y}}{H_{ext}+H_{k}}+R_{ANE}$ (4)

$R_{AD,z}=R_{P}\frac{H_{AD,z}}{H_{ext}}$ (5)

$R_{FL,x}=R_{P}\frac{H_{FL,x}}{H_{ext}}$ (6)

$R_{FL,y}=R_{P}\frac{H_{FL,y}+H_{Oe}}{H_{ext}}$ (7)

$R_{FL,z}={\frac{1}{2}R}_{A}\frac{H_{FL,z}}{H_{ext}+H_{k}}$ (8)

and

$\theta_{T,i}=\frac{2eM_{s}t_{FM}}{\hbar}\frac{H_{T,i}}{J_{[Pt/Co]}}$ (*T*=AD, FL; *i*=*x*, *y*, *z*) (9)

where *R*_ANE_ is the anomalous Nernst resistance, which can be separated from SOT contributions by fitting *H*_ext_-dependent *R*_AD,y_ using Supplementary Eq. (4) after extracting the *R*_AD,y_ component from *R*_H,2ω_ measurement using Supplementary Eq. (2). *H*_k_ is the magnetic anisotropic field depending on the Co thickness, which is extracted out from the out-of-plane field sweep of *R*_H,1ω_. *H*_Oe_ is the current-induced magnetic field in the transverse direction mixing into the term of *H*_FL,y_, can be approximated by μ_0_*I*/2*w*, where *I* is the charge current through underlayer, and *w* is the width of current channel. *H_T_*_,_*_i_* and θ*_T_*_,_*_i_* (*T*=AD, FL; *i*=*x*, *y*, *z*) are the spin-orbit effective fields and spin-torque efficiencies respectively corresponding to the spin-orbit torques τ*_T_*_,_*_i_* (*T*=AD, FL; *i*=*x*, *y*, *z*). *H_T_*_,_*_i_* (*T*=AD, FL; *i*=*x*, *y*, *z*) is extracted by fitting Supplementary Eqs. (3)-(8) in dependency of 1/*H_ext_* or 1/(*H_ext_*+ *H_k_*). The parameters *e*, *M*_s_, *t*_FM_, *ħ*, and *J*_[Pt/Co]_ in Supplementary Eq. (9) are electron charge, saturation magnetization and thickness of the CoFeB free layer, reduced Planck’s constant, and current density flowing through [Pt/Co]_N_, respectively. The *J*_[Pt/Co]_ was estimated considering the shunting effect by using the parallel resistor model. Furthermore, the reported contribution of ordinary Nernst effect (ONE) [5] to the *R*_H,2ω_ was also confirmed to be absent in our sample stacks [4].

**ST-FMR characterizations on in-plane magnetized samples.** ST-FMR measurements were performed on in-plane magnetized film stacks in patterned rectangular strips with the size of 20 μm by 30 μm. The external field *H*_ext_ is generated by a set of magnetic coils which can go up to 2.6 kOe. The field is oriented with a 45^o^ angle with respect to the strip thus the current flow direction. A GHz signal (2-20 GHz) with a fixed power was generated by an HP 83624B microwave source and was applied through a T-Bias and ground-signal-ground coplanar waveguide. The GHz signal injected into [Pt/Co]_N_ generates oscillating spin current flowing along ± *z*-axis, which exert spin-orbit torques τ*_T_*_,_*_i_* (*T*=AD, FL; *i*=*x*, *y*, *z*) and Oe-field induced torque τ_Oe_ on the CoFeB in-plane magnetization. The product of the injected rf current and oscillating resistance produces a mixing voltage (*V*_mix_) which can be decoupled into a symmetric part and an asymmetric part, as shown in Supplementary Fig. 3a. The symmetric part (*S*) of the *V*_mix_ corresponds to the toques from τ_AD,_*_x_*, τ_AD,_*_y_*, and τ_FL,_*_z_*, and the asymmetric part (*A*) of that consists of τ_FL,_*_x_*, τ_FL,_*_y_*, τ_AD,_*_z_*, and τ_Oe_ [6]. The charge-to-spin conversion efficiency θ_AD_ can be evaluated by using the simplified relation [7]:

$\theta_{AD}=\frac{S}{A}\frac{eM_{s}t_{FM}t_{[Pt/Co]}}{\hbar}\sqrt{1+\frac{M_{eff}}{H_{0}}}$ (10)

where$H_{0}$is the resonance field, *M*_eff_ is the effective demagnetization. The model presented in Supplementary Eq. (10) can approximate the conventional spin efficiency as τ_AD,_*_y_* and τ_Oe_ are predominant in the components of *S* and *A*, respectively. The symmetric and asymmetric Lorentzian functions given by Supplementary Eq. (11) are used to extract *S*, *A*, *H*_0_, and Δ*H* (full width at half maximum (FWHM)) from the *V*_mix_ signal,

$V_{mix}=\frac{S{\Delta H}^{2}}{{\Delta H}^{2}+{(H_{ext}-H_{0})}^{2}}+\frac{A(H_{ext}-H_{0})\Delta H}{{\Delta H}^{2}+{(H_{ext}-H_{0})}^{2}}$ (11)

The ST-FMR measurement was performed under constant 20 dBm power with the excitation frequency from 3 to12 GHz. The *M*_eff_ is obtained by fitting the excitation frequency *vs*. *H*_0_ using the Kittel formula

$f=\frac{\gamma}{2\pi}\sqrt{H_{0}(H_{0}+4\pi M_{eff})}$ (12)

where γ is the gyromagnetic ratio. By linear fitting of Δ*H* dependent on resonant frequency *f*,

$\Delta H={\Delta H}_{0}+\frac{4\pi}{\sqrt{3}\gamma}\alpha f$ (13)

we obtained the damping constant α of the magnetic layer. Here, Δ*H*_0_ is the linewidth due to film inhomogeneity broadening [8]. In the Si/SiO_2_/SRT1/Mg(2)/CoFeB(2.5)/MgO(1.5)/Ta(2) sample, the charge-to-spin conversion efficiency θ_AD_ is calculated to be 0.082 ± 0.013 on average from 3 to 12 GHz, the effective demagnetization *M*_eff_ = 0.944 ± 0.011, and damping constant α= 0.0088 ± 0.0003. The ST-FMR measurement on SRT2-based samples shows much broader resonance in far-beyond the applicable external magnetic fields in the range of 3-12 GHz current excitation. This is due to the strong perpendicular magnetic anisotropy in SRT2 stacks which requires in-plane magnetic fields larger than *H*_k_ to orientate the spins precession along in-plane direction with a large magnetic damping constant.

**Current-dependent second harmonic Hall (SHH) measurement for SOT characterization.** For samples with negligible SOTs from unconventional polarizations σ*_x_* and σ*_z_* compared to those from conventional polarizations σ*_y_*, there is a model named as current-dependent SHH, simplified from the angular-dependent SHH as discussed above. In this simplified model, the expression of the second harmonic Hall resistance *R*_H,2ω_ can be rewritten from Supplementary Eqs. (2), (4), and (7) as

$R_{H,2\omega}=\left( {\frac{1}{2}R}_{A}\frac{H_{AD,y}}{H_{ext}+H_{k}}+R_{ANE} \right)\cos\varphi+R_{P}\frac{H_{FL,y}+H_{Oe}}{H_{ext}}cos2\varphi\cos\varphi$ (14)

In Supplementary Eq. (14), when the external in-plane magnetic field overcomes the uniaxial magnetic anisotropy to align the magnetization and the field angle ϕ is fixed at 45^o^, the cos2*ϕ*cos*ϕ* term could be excluded. Further, in the cos*ϕ* term, only the damping-like field depends on the external magnetic field. By sweeping the magnitude of external fields in 45^o^ and plotting *R*_H,2ω_ as a function of 1/(*H*_ext_*+H*_k_), we can determine the values of *H*_AD,_*_y_* and *R*_ANE_ by linear fitting. The change of current amplitude *I* tunes the current-induced damping-like effective field correspondingly. The field-sweep measurement and linear fitting of *R*_H,2ω_ vs. 1/(*H*_ext_*+H*_k_) extract out *H*_AD,_*_y_* at different current amplitude *I*; and by linear fitting of *H*_AD,_*_y_* vs. *I* as shown in Supplementary Fig. 3b, we calculated the spin efficiency based on Supplementary Eq. (9) at *T*=AD and *i*=*y*.

**Differential planar Hall effect (DPHE) measurement for in-plane type-x SOT switching.** The DPHE method is based on the dependence of the PHE signal with respect to the angular orientation of in-plane ***M***, which is proportional of sin2*ϕ* from Supplementary Eq. (1). When magnetic anisotropy is along *x* axis, the addition of a small bias field *H_y_* << *H*_k_ will result in a small in-plane tilt of ***M***, defining as Δ*ϕ*. Considering the four situations of PHE signals when ***M*** is along +*x* or -*x* with bias field *H_y_* along +*y* or -*y*:

$R_{H}\left( M_{+x},H_{+y} \right)=\frac{R_{P}}{2}\sin(2\Delta\varphi)$ (15)

$R_{H}\left( M_{+x},H_{-y} \right)=\frac{R_{P}}{2}\sin(-2\Delta\varphi)$ (16)

$R_{H}\left( M_{-x},H_{+y} \right)=\frac{R_{P}}{2}\sin2({180}^{o}-\Delta\varphi)$ (17)

$R_{H}\left( M_{-x},H_{-y} \right)=\frac{R_{P}}{2}\sin2({180}^{o}+\Delta\varphi)$ (18)

Derived from Supplementary Eqs. (15)- (18), the DPHE signals at *M*(+*x*) and *M*(-*x*) are

${\Delta R}_{H}\left( M_{+x} \right)=R_{H}\left( M_{+x},H_{+y} \right)-R_{H}\left( M_{+x},H_{-y} \right)=R_{P}\sin(2\Delta\varphi)$ (19)

${\Delta R}_{H}\left( M_{-x} \right)=R_{H}\left( M_{-x},H_{+y} \right)-R_{H}\left( M_{-x},H_{-y} \right)=-R_{P}\sin(2\Delta\varphi)$ (20)

Therefore, the DPHE signals show opposite sign when the magnetization aligns in +*x* or -*x* direction, which can be an indicator of the magnetization switching along *x* direction. Similarly, the DPHE method also works for detecting magnetization switching along *y* direction, in which the small bias field *H*_x_ is applied in *x* direction. An alterantive method of detecting in-plane magentization switching with similar principle is to use sinusoidal read current without external bias field [9].

In the field-sweep measurement strategy as shown in Supplementary Fig. 4c, an in-plane magnetic field *H_x_* along *x* direction is generated by a set of coils without magnet cores actuated by Kepco Power Supply by current control. When *H_x_* is off, we read the PHE signals with a 400-μA read current *I_R_* from a Keithley 220 current source. During PHE signal read, a small bias field *H_y_* generated by another set of coils without magnet cores is applied along +*y* and -*y* directions by turns, so that we can read PHE voltages of both *V_H_*(*H_+y_*) and *V_H_*(*H_-y_*) by turns using a Keithley 2000 multimeter. The two sets of coils composed into a vector-field structure with *x* and *y* directions. The coils are purposely designed without magnet cores sacrificing large magnetic fields to guarantee no remanent fields so that the controlling current is proportional of the magnetic field, which is precisely required for the small bias fields. As the measurement strategy shown in Supplementary Fig. 4c, the current-induced switching measurement uses a write current pulse *I*_W_ with 1ms width from the Keithley 220 current source to replace the magnetic field *H_x_* comparing to the field-sweep measurement. The PHE signal read strategy is the same for both field-sweep and current-induced switching measurements.

In Supplementary Figs. 5a and 5b, we presented the field-sweep and current-induced switching measurements on the sample of SRT1/Mg(2)/CoFeB(2.5)/MgO(1.5)/Ta(2) deposited on Si/SiO_2_ substrates with magnetic anisotropy along *x* direction using DPHE method, as a performance comparison of the same sample deposited on (100) MgO single crystal substrates in Fig. 3b and 3c in the main manuscript. In this sample, the in-plane spin efficiencies are θ_AD,x_= -0.0190± 0.004, and θ_AD,y_= 0.1059± 0.006. Consistent with the micromagnetic simulation, 20% of *x*-polarized spin over *y*-polarized spin is large enough for a field-free type-*x* SOT switching. In Supplementary Fig. 5b, the current-induced switching curve indicates a sharp and full switch from *M_-x_* to *M_+x_* at -30 mA with the coercivity of 5.2 Oe; while the opposite switching from *M_+x_* to *M_-x_* is not fully finished at +26 mA and stay between -*x* axis and *y* axis. This could result from the larger coercivity of 13.0 Oe and pinning domains compared to the opposite switching, or insufficient SOTs from *x*-polarized spins at such a current amplitude.

**Type-y SOT switching measurement by USMR method.** For in-plane type-y magnetization switching detection, magnetic tunneling junction (MTJ) device is generally used. The study of spin Hall magnetoresistance (SMR) in HM/FM bilayers [10] found strong signals in the CoFeB-based system, and the unidirectional SMR (USMR) [11,12] mechanism is further used to detect the in-plane magnetization switching along *y* direction using a Hall-bar device. In the case of in-plane magnetized CoFeB as a ferromagnetic layer, the second harmonic of longitudinal resistance *R_xx_*_,2ω_ at high and low states are observed when CoFeB magnetization *M* and spin magnetic moment σ*_y_* are parallel and anti-parallel, respectively. Supplementary Fig. 5c shows a field-sweep measurement of *R_xx_*_,2ω_ in a Hall-bar device. The field is applied along *y* direction, and the sample is SRT1/Mg(2)/CoFeB(2.5)/MgO(1.5)/Ta(2) deposited on Si/SiO_2_ substrates with magnetic anisotropy in *y* direction. The ac current for this measurement is with the magnitude of 2 mA and the frequency of 1.33 kHz. The in-plane coercivity of this 10 μm-width Hall-bar device is ~ 4 Oe. For the current-induced type-y SOT switching of the in-plane CoFeB magnetization (Supplementary Fig. 5d), we use 1ms-width ac current pulse. The magnetization is initialized along either –*y* or +*y* direction using strong external magnetic fields. The read current keeps the same settings as of those in the field-sweep USMR measurement. The *R_xx_*_,2ω_ at high and low states in current-induced switch is consistent with those at zero field in the field-sweep curve, indicating a full type-y switching in this sample. The switching current of *M_-y_* -> *M_+y_* and *M_+y_* -> *M_-y_* is at +20 mA and -20mA, respectively. In this sample, the in-plane spin efficiencies are θ_AD,x_= -0.0190± 0.004, and θ_AD,y_= 0.1059± 0.006. As *x*-polarized spin efficiency is about 20% of *y*-polarized spin efficiency, it is reasonable to see that the switching current in the type-y configuration (Supplementary Fig. 5d) is smaller than that in the type-x (Supplementary Fig. 5b). The different switching current also excluded the possibility of type-xy switching configuration [13].

**Micromagnetic switching simulations in type-x and type-y configurations.** The micromagnetic simulation utilizes an open-source software Mumax3, where we initialize the magnetization of an ellipse with the aspect ratio of 3 in –*x* or –*y* direction for type-x and type-y devices, respectively, and switch the magnetization by applying a charge current that can be converted into polarized spins and thereby generates SOTs. The SOT is implemented by

$\tau_{SOT}=J_{c}\frac{\hbar}{2eM_{s}t_{FM}}\left[ \theta_{SH}\boldsymbol{m}\times\left( \boldsymbol{m}\times\boldsymbol{\sigma}_{\boldsymbol{AD}} \right)+\theta_{SH}\boldsymbol{m}\times\boldsymbol{\sigma}_{\boldsymbol{FL}}) \right]$ (21)

where *J*_c_ is the charge current, $\hbar$ is the reduced Planck’s constant, *e* is the charge of an electron, θ_SH_ is the SOT efficiency, *M*_s_ is the saturation magnetization, *t*_FM_ is the free layer thickness, ***m*** is the magnetization vector, **σ_AD_** is the damping-like torque vector, and **σ_FL_** is the field-like torque vector. Simulation volume is an ellipse with dimensions of 128$\times$384$\times$2.5 nm^3^ which is placed along either *x* or *y* direction, where the charge current flows along the +*x* direction. The elliptical free layer has the saturation magnetization of 1.2x10^6^ A/m, the exchange coupling of 12 pJ/m^3^, the anisotropy constant of 300 kJ/m^3^, and the damping constant of 0.0088. The size of the simulation unit cell is 6$\times$6$\times$2.5 nm^3^. The SOT efficiency and vector parameters are set as θ_SH_σ_AD_= (θ_AD,_*_x_*, θ_AD,_*_y_*, 0) and θ_SH_σ_FL_= (0, θ_FL,_*_y_*, 0). The charge current, or the SOTs, is applied for a certain width of *t*_pulse_ and the magnetization is relaxed to determine whether the FM layer is deterministically switched. Switching current and current density are determined by sweeping the charge current, *t*_pulse_, and σ_AD,_*_x_*/σ_AD,_*_x_*.

**Supplementary Figures**


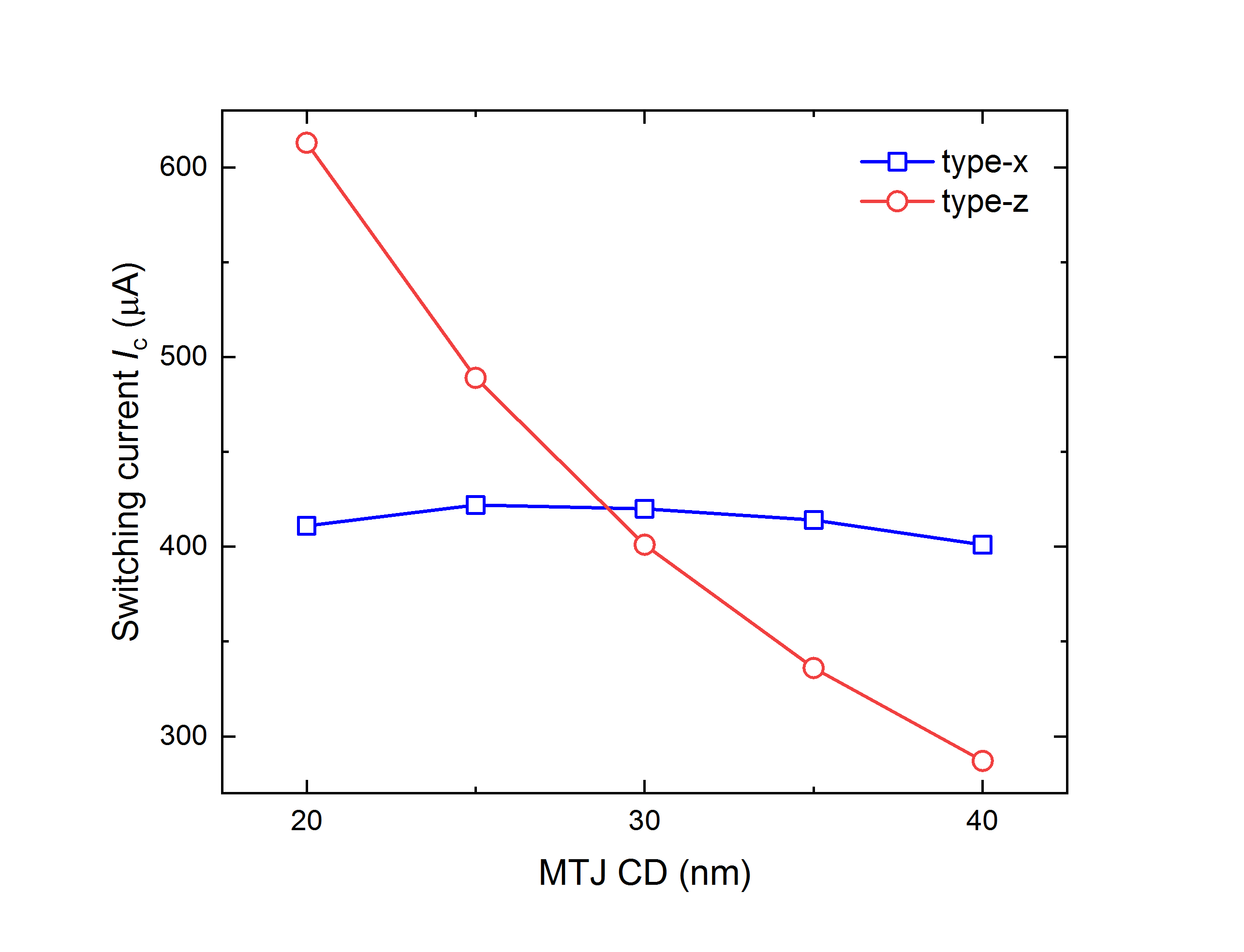


**Supplementary Fig. 1: Critical switching current *I*_c_ in type-x and type-z SOT configurations as a function of MTJ CD**. In performing micromagnetic simulations in both configurations, external field is assumed to be 120 mT, thermal stability ratio Δ is kept at a constant of 60 at 350 K, and the current pulse width is 0.8 ns. In the type-x MTJ cell, in-plane aspect ratio (AR) of the free layer (FL) is 3; in the type-z MTJ cell, the FL thickness is fixed at 1 nm.


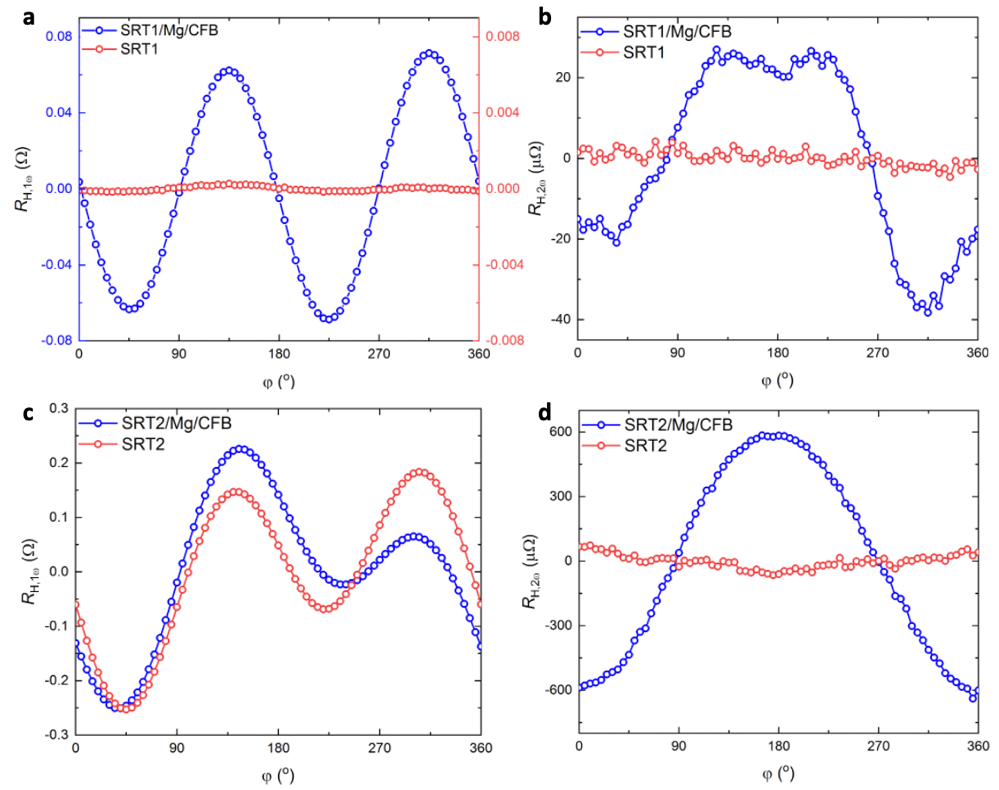


**Supplementary Fig. 2: Angular-dependent SHH signals. a,c** *R*_H,1ω_ v.s. ϕ; **b,d** R_H,2ω_ v.s. ϕ. In-plane field *H*_ext_=200 mT. Samples are SRT1, SRT1/Mg/CFB, SRT2, SRT2/Mg/CFB deposited on Si/SiO_2_.


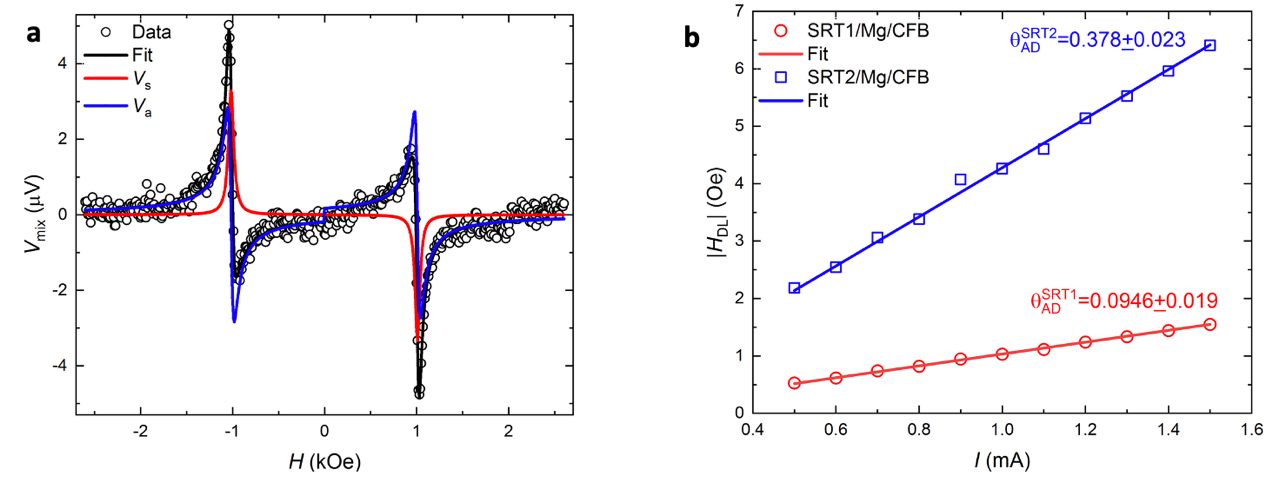


**Supplementary Fig. 3: SOT characterization using ST-FMR and current-dependent SHH techniques on Si/SiO_2_/SRT1 (or SRT2)/Mg(2)/CoFeB(2.5)/MgO(1.5)/Ta(2) samples: a,** the ST-FMR DC voltage signal *V*_mix_ *vs*. in-plane magnetic field *H* with excitation current at 9 GHz on the SRT1/Mg/CoFeB sample. **b**, the current-dependent damping-like toque effective field *H*_DL_ and linear fittings on the SRT1/Mg/CoFeB and SRT2/Mg/CoFeB samples.

**
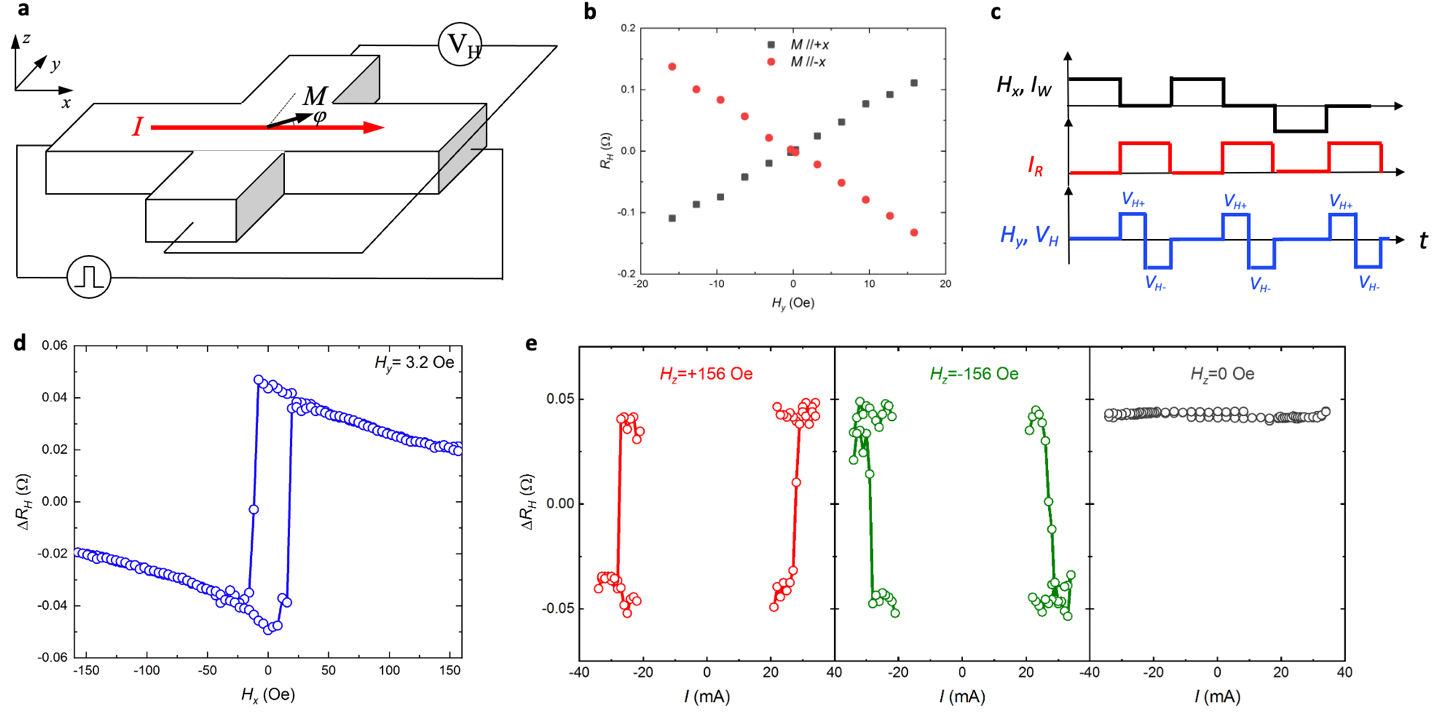
**

**Supplementary Fig. 4: DPHE measurement methodology and SOT switching in type-x configuration on a reference sample Pt/Mg/CoFeB: a**, A schematic depiction of a Hall-bar for studying type-x SOT switching. ***M*** is the magnetization of free layer and the easy axis of the free layer is oriented along the longitudinal current direction. **b**, *R*_H_ *vs. H_y_* bias measured at *M* along +*x* and at *M* along -*x*. *R*_H_ = *V*_H_ /*I*. **c**, A description of the measurement sequence for field-switching and current-switching using DPHE method, with *x* in the longitudinal direction and *y* in the transverse direction. *I*_W_ and *I*_R_ represent the writing current for current-induced SOT switching, and the reading current for *R*_H_ sensing. Both *I*_W_ and *I*_R_ are applied along *x* direction. *I*_R_=2.0 mA for the 10μm-width Hall-bar device. **d,e,** The DPHE curves measured for field-switching (**d**) and current-switching (**e**) in type-x SOT configuration in Pt(3)/Mg(2)/CoFeB(2.5)/MgO(1.5)/Ta(2) samples. The bias field *H_y_* is 3.2 Oe. The current-switching is assisted by different external field *H_z_* (+156 Oe, -156 Oe, and 0 Oe) in the plot.


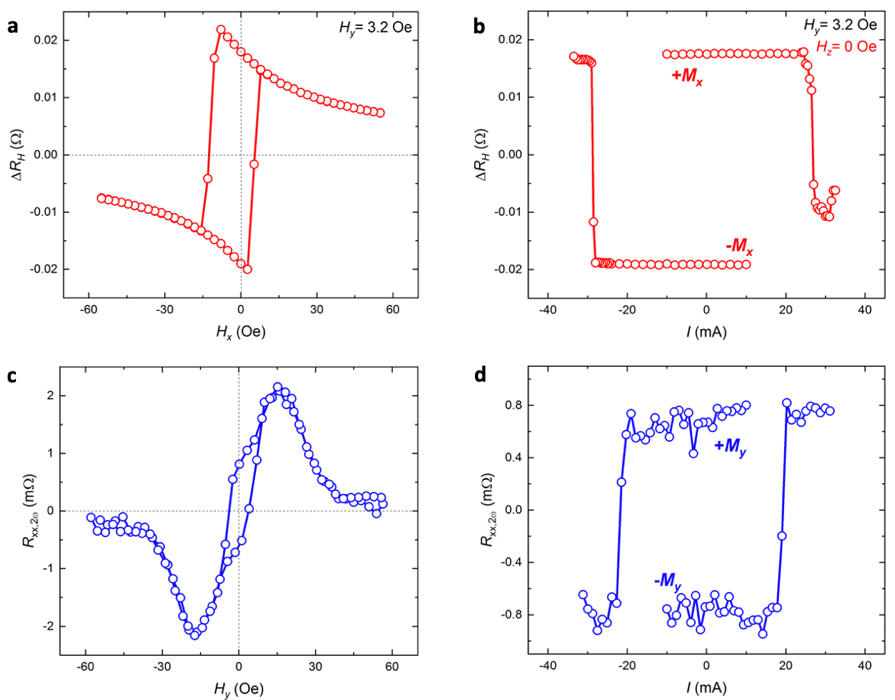


**Supplementary Fig. 5: Type-x and type-y SOT switching behaviors in the SRT1/Mg/CoFeB samples deposited on Si/SiO_2_.** **a**,**b**, field-sweep switching and field-free current-induced switching in type-x configuration based on DPHE measurement in SRT1/Mg(2)/CoFeB(2.5)/MgO(1.5)/Ta(2) deposited on Si/SiO_2_ substrate with magnetic anisotropy in *x* direction. **c**,**d**, field-sweep switching and field-free current-induced switching in type-y configuration based on USMR measurement in SRT1/Mg(2)/CoFeB(2.5)/MgO(1.5)/Ta(2) deposited on Si/SiO_2_ substrate with magnetic anisotropy in *y* direction.


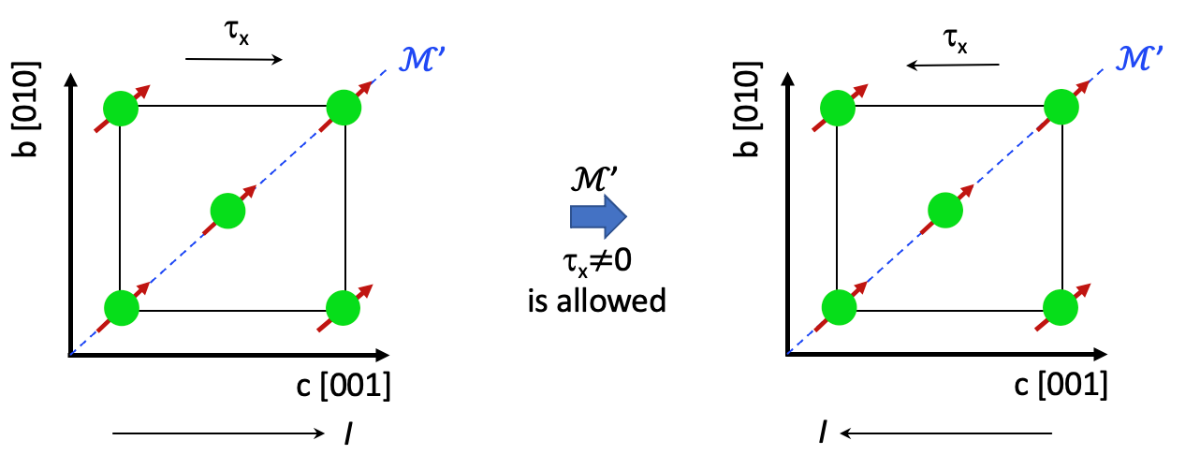


**Supplementary Fig. 6: An illustration of symmetry breaking in weakly in-plane magnetized low-dimensional Co sputtered on Pt films.** The *bc*-plane is parallel to the film plane, the current *I* is applied along the *c*-axis, and the unconventional spin-orbit torque τ*_x_* from *x*-polarized spins is along the *c*-axis, too. The green spheres and red arrows represent the Co atoms and the magnetic moment, respectively. The blue dashed line represents the magnetic mirror symmetry $\mathcal{M'}$, under which, the unconventional spin-orbit torque τ*_x_* is allowed.

**
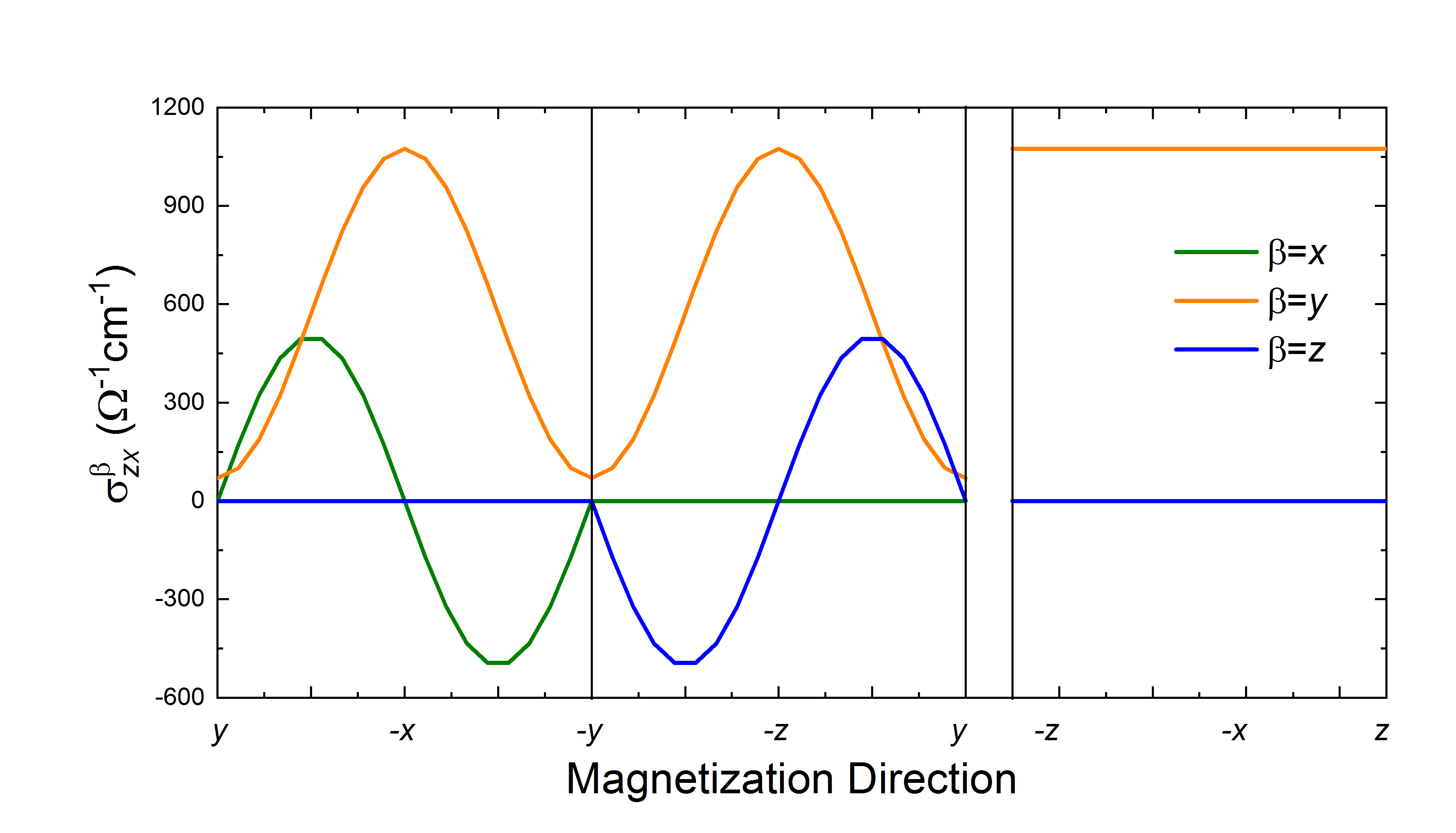
**

**Supplementary Fig. 7: Magnetization dependence of intrinsic spin Hall conductivity σ^β^*_zx_* of Co (hcp).** The charge current is applied along *x* direction while spin current flows in *z*. The lattice constant c of Co is parallel with the charge current. The horizontal axis gives the magnetization direction, which is swept in the xy, yz, and zx planes. Curves with different colors show spin Hall conductivity with different spin polarizations, notated as β=*x*, *y*, *z*. These curves are calculated from Eq. (2) in the main manuscript. The parameters σ_SHE_ and σ_SAHE_ in Eq. (2) are extracted from the first-principles results as described in reference [14].

**Supplementary References**

1. MacNeill, D., Stiehl, G. M., Guimaraes, M. H. D., Buhrman, R. A., Park, J., and Ralph, D. C. Control of spin–orbit torques through crystal symmetry in WTe_2_/ferromagnet bilayers. *Nat. Phys.* **13**(3), 300-305 (2017).

2. Avci, C. O., Garello, K., Gabureac, M., Ghosh, A., Fuhrer, A., Alvarado, S. F., and Gambardella, P. Interplay of spin-orbit torque and thermoelectric effects in ferromagnet/normal-metal bilayers. *Phys. Rev. B* **90**(22), 224427 (2014).

3. Wen, Y., Wu, J., Li, P., Zhang, Q., Zhao, Y., Manchon, A., ... and Zhang, X. Temperature dependence of spin-orbit torques in Cu-Au alloys. *Phys. Rev. B* **95**(10), 104403 (2017).

4. DC, M., Shao, D-F., Hou, V.D.H, Quarterman, P., Habiboglu, A, … and Wang, S.X. Observation of anti-damping spin-orbit torques generated by in-plane and out-of-plane spin polarizations in MnPd_3_, *Nat. Mat*. 1-8 (2023).

5. Roschewsky, N., Walker, E. S., Gowtham, P., Muschinske, S., Hellman, F., Bank, S. R., and Salahuddin, S. Spin-orbit torque and Nernst effect in Bi-Sb/Co heterostructures. *Phys. Rev. B* **99**(19), 195103 (2019).

6. Chen, X., Shi, S., Shi, G., Fan, X., Song, C., Zhou, X., ... and Pan, F. Observation of the antiferromagnetic spin Hall effect. *Nat. Mat*. **20**(6), 800-804 (2021).

7. Liu, L., Moriyama, T., Ralph, D. C., and Buhrman, R. A. Spin-torque ferromagnetic resonance induced by the spin Hall effect. *Phys. Rev. Lett.* **106**(3), 036601 (2011).

8. Chang, H., Li, P., Zhang, W., Liu, T., Hoffmann, A., Deng, L., and Wu, M. Nanometer-thick yttrium iron garnet films with extremely low damping. *IEEE Magn. Lett.*, 5, 1-4 (2014).

9. Huynh Duy Khang, N., and Hai, P. N. Spin-orbit torque as a method for field-free detection of in-plane magnetization switching. arXiv-2010 (2020).

10. Kim, J., Sheng, P., Takahashi, S., Mitani, S., Hayashi, M. Spin Hall magnetoresistance in metallic bilayers. *Phys. Rev. Lett*. 116, 097201 (2016).

11. Avci, C. O., Garello, K., Ghosh, A., Gabureac, M., Alvarado, S. F., and Gambardella, P. Unidirectional spin Hall magnetoresistance in ferromagnet/normal metal bilayers. *Nat. Phys.* **11**(7), 570-575 (2015).

12. Liu, Y. T., Chen, T. Y., Lo, T. H., Tsai, T. Y., Yang, S. Y., Chang, Y. J., ... and Pai, C. F. Determination of spin-orbit-torque efficiencies in heterostructures with in-plane magnetic anisotropy. *Phys. Rev. Appl.* **13**(4), 044032 (2020).

13. Kong, W. J., Wan, C. H., Wang, X., Tao, B. S., Huang, L., Fang, C., ... and Han, X. F. Spin–orbit torque switching in a T-type magnetic configuration with current orthogonal to easy axes. *Nat. Comm.* **10**(1), 1-7 (2019).

14. Amin, V. P., Li, J., Stiles, M. D., and Haney, P. M. Intrinsic spin currents in ferromagnets. *Phys. Rev. B* **99**(22), 220405 (2019).
